# Global epidemiology of thyroid cancer: trends in incidence, mortality, and DALYs from 1990 to 2021

**DOI:** 10.1515/med-2025-1322

**Published:** 2026-01-30

**Authors:** Wanlin Lei, Jianling Qiang, Chenchen Yi, Maofeng Wang

**Affiliations:** Department of Biomedical Sciences Laboratory, Affiliated Dongyang Hospital of Wenzhou Medical University, Dongyang, Zhejiang, China; Zhejiang Provincial Key Laboratory of Medical Genetics, College of Laboratory Medicine and Life Sciences, Wenzhou Medical University, Wenzhou, Zhejiang, China

**Keywords:** thyroid cancer, global burden of disease, socio-demographic index, DALYs, epidemiology

## Abstract

**Objectives:**

This study provides the first comprehensive assessment of the global thyroid cancer burden from 1990 to 2021, focusing on incidence, mortality, and DALYs trends across 204 countries.

**Methods:**

Using Global Burden of Disease (GBD) 2021 data, age-standardized rates (ASRs) and estimated annual percentage changes (EAPCs) were calculated. Associations with the Socio-demographic Index (SDI) were analyzed via linear regression.

**Results:**

Globally, the age-standardized incidence rate increased from 2.06 to 2.91 per 100,000 (EAPC=1.25), with women accounting for 67 % of cases. Mortality showed a modest decline (EAPC=−0.23), but DALYs remained high (14.57 million in 2021). High-SDI regions, such as North America, accounted for 72 % of cases, reflecting intensive screening, whereas low-SDI regions, particularly Sub-Saharan Africa, contributed 68 % of deaths due to delayed diagnosis. High BMI contributed 1.68 % of DALYs, peaking at 4.26 % in Andean Latin America. Incidence varied significantly across countries, from 7.13 per 100,000 in Saudi Arabia (linked to iodine excess) to 0.014 per 100,000 in Tajikistan (iodine deficiency). Mortality was highest among elderly males, reaching 12.57 per 100,000 in those aged ≥90 years.

**Conclusions:**

The rising global burden of thyroid cancer highlights pronounced gender and regional disparities. High-SDI regions should prioritize risk-stratified strategies to reduce overdiagnosis, while low-SDI regions require improved access to healthcare. Targeted efforts in early detection and metabolic risk reduction are critical to mitigate disease burden.

## Introduction

The global incidence of thyroid cancer has been steadily increasing. Although ultrasound examination is effective in classifying cancer nodules for biopsy, its relatively low specificity has contributed to the problem of overdiagnosis [1], [2], [3], [4]. Epidemiological data indicate that the incidence of thyroid cancer in both men and women shows an initial slight decline, followed by a rise, and eventually a significant increase, with women being affected approximately three times more frequently than men [5], [6], [7]. Surgical resection remains the principal treatment modality for thyroid cancer; however, the widespread application of diagnostic methods such as ultrasound has exacerbated overdiagnosis. Concurrently, the limited specificity of these techniques often results in unnecessary surgeries and treatments. Conventional treatments, such as surgery and radioactive iodine therapy, may induce adverse effects, including postoperative complications and hypothyroidism after radioactive iodine treatment [8], [9], [10], [11], [12], [13]. Recently, the emergence of new treatment methods, such as targeted therapy and immunotherapy, has expanded the therapeutic armamentarium for patients [14]. However, these strategies may also be associated with adverse outcomes, including immune-related adverse events. Currently, the early diagnosis of thyroid cancer relies predominantly on imaging methods, such as ultrasound [15]. However, these techniques exhibit limited sensitivity and specificity for detecting early micro-lesions, and definitive guidelines for the diagnosis and management of undifferentiated thyroid cancer remain lacking. Collectively, these characteristics highlight the epidemiological patterns and temporal trends of thyroid cancer, which may not only aid in predicting future trends but also support the Chinese government in formulating effective health policies [16], [17], [18].

## Methods

### Characteristics of the data source

This cross-sectional study employed the Global Health Data Exchange query tool, created by the GBD collaborators, to collect standardized age-specific data on thyroid cancer, including disease definitions and prevalence information. The Global Burden of Disease 2021 project provides estimates of morbidity, mortality, and disability-adjusted life years (DALYs) for 369 diseases and injuries across 204 countries and territories from 1990 to 2021. For this study, annual incidence, prevalence, and mortality rates of thyroid cancer, as well as corresponding age-standardized rates (ASRs) stratified by sex, region, country, and etiology, were extracted from the Global Health Data Exchange query tool for the period 1990–2021 (http://ghdx.healthdata.org/gbd-results-tool). This study was conducted in accordance with the Strengthening the Reporting of Observational Studies in Epidemiology (STROBE) guidelines.

### Sociodemographic index

The Socio-Demographic Index (SDI) is a quantitative measure of the level of development of a country or region, incorporating indicators of fertility, education, and per capita income [19]. The SDI is expressed as a continuous variable ranging from 0 to 1, with higher values representing greater levels of socio-economic development. Previous studies have demonstrated associations between SDI and disease morbidity and mortality. In this study, countries and regions were classified into five SDI groups (low, low-medium, medium, medium-high, and high). Moreover, these countries were geographically organized into 21 regions, including the high-income Asia-Pacific and Central Asia. The objective of this study was to investigate the relationship between thyroid cancer burden and socio-economic development.

### Statistical analysis

The primary indicators used to describe thyroid cancer burden were incidence, mortality, DALYs, and their corresponding rates. All rates were reported per 100,000 population, with 95 % uncertainty intervals (UI) calculated according to the GBD algorithm. To evaluate temporal trends in disease burden, estimated average production costs (EAPCs) and their 95 % confidence intervals (CIs) were determined by linear regression models. A decreasing trend was defined as an EAPC and its upper 95 % CI limit below zero, whereas an increasing trend was defined as an EAPC and its lower 95 % CI limit above zero. Gaussian curves were applied to analyze the relationship between thyroid cancer incidence and mortality and the Human Development Index. Potential risk factors for thyroid cancer were also assessed. All statistical analyses were conducted using R Studio, version 4.1.2 (R Project for Statistical Computing). Statistical significance was set at two-sided p<0.05.

## Results

### Thyroid cancer: global trends

#### Incidence

**Figure 1:**
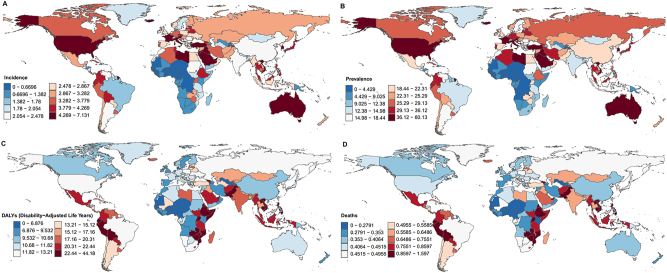
Global trends in thyroid cancer in 2021. (A). Incidence. (B). Prevalence. (C). Deaths. (D). DALYs.

In 2021, there were 249,538 incident cases of thyroid cancer (82,301 in males [33 %] and 167,237 in females [67 %]). Globally, the incidence rate increased from 2.062 (95 % uncertainty interval [UI]: 1.951–2.224) in 1990 to 2.914 (95 % UI: 2.607–3.213) in 2021. The EAPC during this period was 1.250 (95 % confidence interval [CI]: 1.130–1.370) (Table S1-1). Thyroid cancer was consistently more common in women than in men across all age groups. The highest number of incident cases occurred in individuals aged 55–59 years, with 11,441 cases in men (95 % UI: 13,132–5,759) and 19,601 cases in women (95 % UI: 22,578–17266). The highest age-specific incidence rate was observed among women aged 70–74 years (10.872; 95 % UI: 12.370–9.519) and men aged 85–89 years (8.265; 95 % UI: 9.122–7.015) (Figure 1A).

#### Prevalence

In 2021, the global prevalence of thyroid cancer was 1,987,148 cases, comprising 627,658 males (32 %) and 1,359,490 females (68 %). The prevalence rate in 2021 was 23.143 (95 % UI: 20.663–25.647), a significant increase from 14.931 (95 % UI: 14.124–16.029) in 1990, with an EAPC of 1.584 (95 % CI: 1.436–1.732) during this period (Table S1-2). The highest number of prevalent cases occurred among individuals aged 55–59 years, with 95,396 cases in men (95 % UI: 109,572–81575) and 170,229 cases in women (95 % UI: 196,310–150283). The highest age-specific prevalence rate was observed in men aged 55–59 years (48.990; 95 % UI: 56.270–41.893) and women aged 60–64 years (85.433; 95 % UI: 97.938–75.048) (Figure 1B).

#### Mortality

The global mortality rate of thyroid cancer declined slightly from 0.570 (95 % UI: 0.530–0.628) in 1990 to 0.530 (95 % UI: 0.470–0.575) in 2021. The EAPC was −0.232 (95 % CI -0.251 to −0.213), confirming a decrease in death rate over the 30-year period (Table S1-3). The greatest number of thyroid cancer-related deaths occurred in men aged 75–79 years (2,577; 95 % UI: 2,937–2,111) and women aged 70–74 years (3,680; 95 % UI: 4,322–3,147). Mortality rates generally increased with age, except in men aged ≥95 years. The highest age-specific mortality rates were observed in women aged≥95 years (8.936; 95 % UI: 9.925–7.446) and men aged 90–95 years (12.570; 95 % UI: 14.773–8.612) (Figure 1C).

#### DALYs

In 2021, the global number of thyroid cancer-associated DALYs was 14.571 (95 % UI: 12.783–16.115), representing a 0.042 % decrease compared with 2019 (15.20; 95 % UI: 14.184–16.830). Over the 30-year period from 1990 to 2021, the EAPC was −0.140 (95 % UI: −0.168 to −0.112) (Table S1-4). In 2021, the highest number of thyroid cancer-associated DALYs was observed in men aged 55–59 years (66,017; 95 % UI: 75,564–53,303) and women aged 65–69 years (89,175; 95 % UI: 103,398–75,121). The greatest age-specific DALY rate in men occurred in those aged 90–94 years (78.667; 95 % UI: 87.225–65.546), while in women the highest rate was observed among those aged ≥95 years (102.529; 95 % UI: 120.252–70.279) (Figure 1D).

### Thyroid cancer: SDI regional trends

#### Incidence

**Figure 2:**
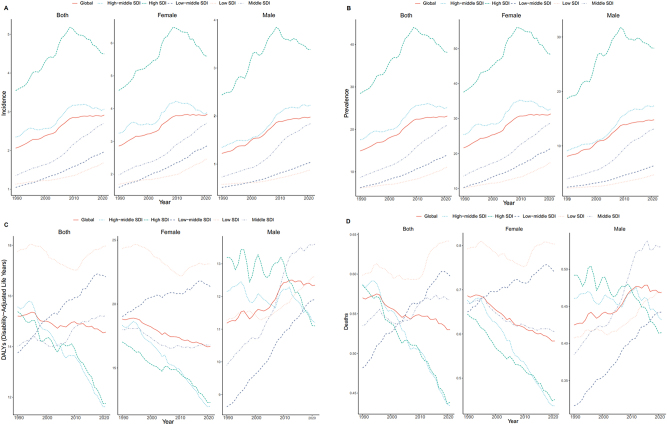
Epidemiologic trends of incidence, deaths, DALYs and prevalence in 5 SDI regions of thyroid cancer from 1990 to 2021. (A). Trends of incidence in 5 SDI regions. (B). Trends of prevalence in 5 SDI regions. (C). Trends of DALYs in 5 SDI regions. (D). Trends of deaths in 5 SDI regions.

**Figure 3:**
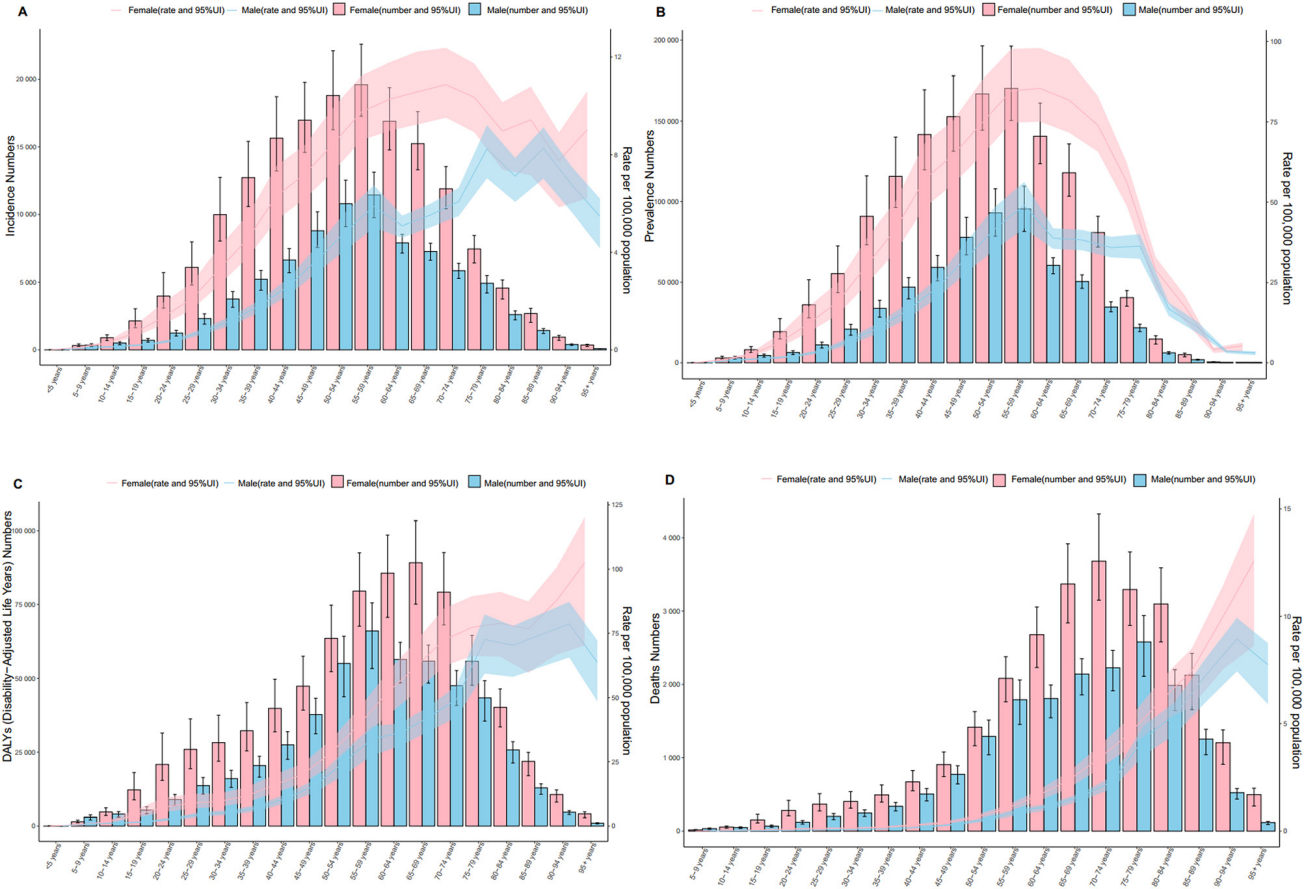
Global trends in thyroid cancer in 2021. (A). Thyroid cancer. (B). Prevalence. (C). DALYs. (D). Deaths.

In 2021, the highest incidence rate was observed in the high-SDI region (4.493; 95 % UI: 4.745–4.252). Over the 30 years, incidence rates in this region exhibited a fluctuating ‘mountain-shaped’ pattern, peaking in 2009 (5.182; 95 % UI: 5.444–4.920) before subsequently declining. In contrast, the lowest incidence rate in 2021 was recorded in the low-SDI region (1.689; 95 % UI: 1.328–2.200). The most pronounced increase occurred in the middle-SDI region, with EAPC 2.373 (95 % CI: 2.280–2.466) (Figure 2A). Across all regions, the highest incidence was observed in patients aged ≥70 years (Figure 3A).

#### Prevalence

In 2021, the highest prevalence rate was observed in the high-SDI region (38.161; 95 % UI: 40.373–36.318). The middle-SDI region had the greatest increase in prevalence rate over the study period, with an EAPC of 3.031 (95 % UI: 2.943–3.119). Overall, prevalence and rates of thyroid cancer were highly correlated with SDI, with higher SDI values corresponding to higher thyroid cancer rates. Gender differences were also evident, as females consistently exhibited higher incidence and prevalence rates than males (Figure 2B). Regarding age distribution, prevalence peaked among individuals aged 50–60 years, exceeding that of other age groups (Figure 3B).

#### DALYs

In 2021, the highest rate of thyroid cancer-associated DALYs was recorded in the low-SDI region (17.976; 95 % UI: 23.060–14.180). Between 1990 and 2021, the DALY rate indicated a downward trend in the high-SDI and high-middle SDI regions, whereas an upward trend was observed in the low-middle SDI region. This pattern may be partly explained by the predominance of female patients, whose DALYs trend mirrored the overall global trend. Among males, marked changes were observed in the middle-SDI and low-middle SDI regions. In the middle-SDI region, the DALYs rate increased from 14.016 (95 % UI: 16.178–12.761) in 1990 to 15.218 (95 % UI: 16.807–12.811) in 2021. Similarly, in the low-middle SDI regions, the DALY rate increased from 13.836 (95 % UI: 17.162–12.073) in 1990 to 16.755 (95 % UI: 19.425–14.349) in 2021 (Figure 2C). Across all SD1 regions, DALY rates were highest among individuals aged≥70 years (Figure 3C).

#### Mortality

Between 1990 and 2021, death rates from thyroid cancer were always highest in low-SDI regions. In this region, the lowest mortality was observed in 2009 (0.593; 95 % UI: 0.724–0.476), followed by an increase to 0.642 (95 % UI: 0.799–0.516) in 2021. Over the same period, mortality rates declined steadily in high-SDI and high-middle-SDI regions, but increased in middle-SDI and low-SDI regions. Globally, the thyroid cancer mortality rate demonstrated a gradual downward trend. Sex-specific patterns were also evident: mortality among females declined consistently, whereas male mortality fluctuated, but showed an overall decline between 2015 and 2021 (Figure 2D). Across age groups, the highest death rates were observed among individuals aged 50–69 years (Figure 3D).

### Thyroid cancer: geographic regional trends

#### Incidence

**Figure 4:**
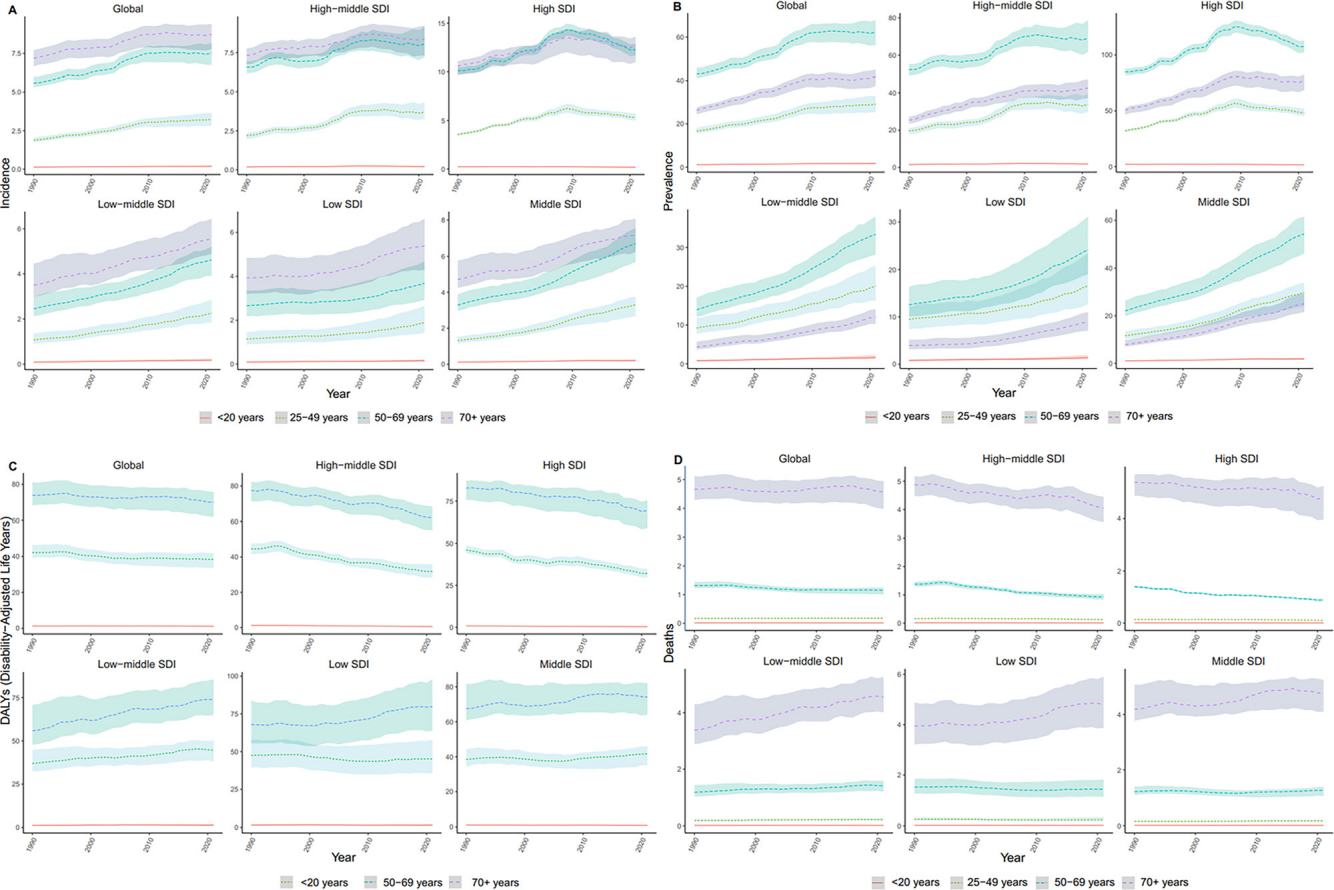
The incidence, mortality, DALYs and prevalence of thyroid cancer in different age groups in five SDI regions from 1990 to 2021. (A). Trends of incidence different age groups in 5 SDI regions. (B). Trends of prevalence different age groups in 5 SDI regions. (C). Trends of DALYs different age groups in 5 SDI regions. (D). Trends of deaths different age groups in 5 SDI regions.

**Figure 5:**
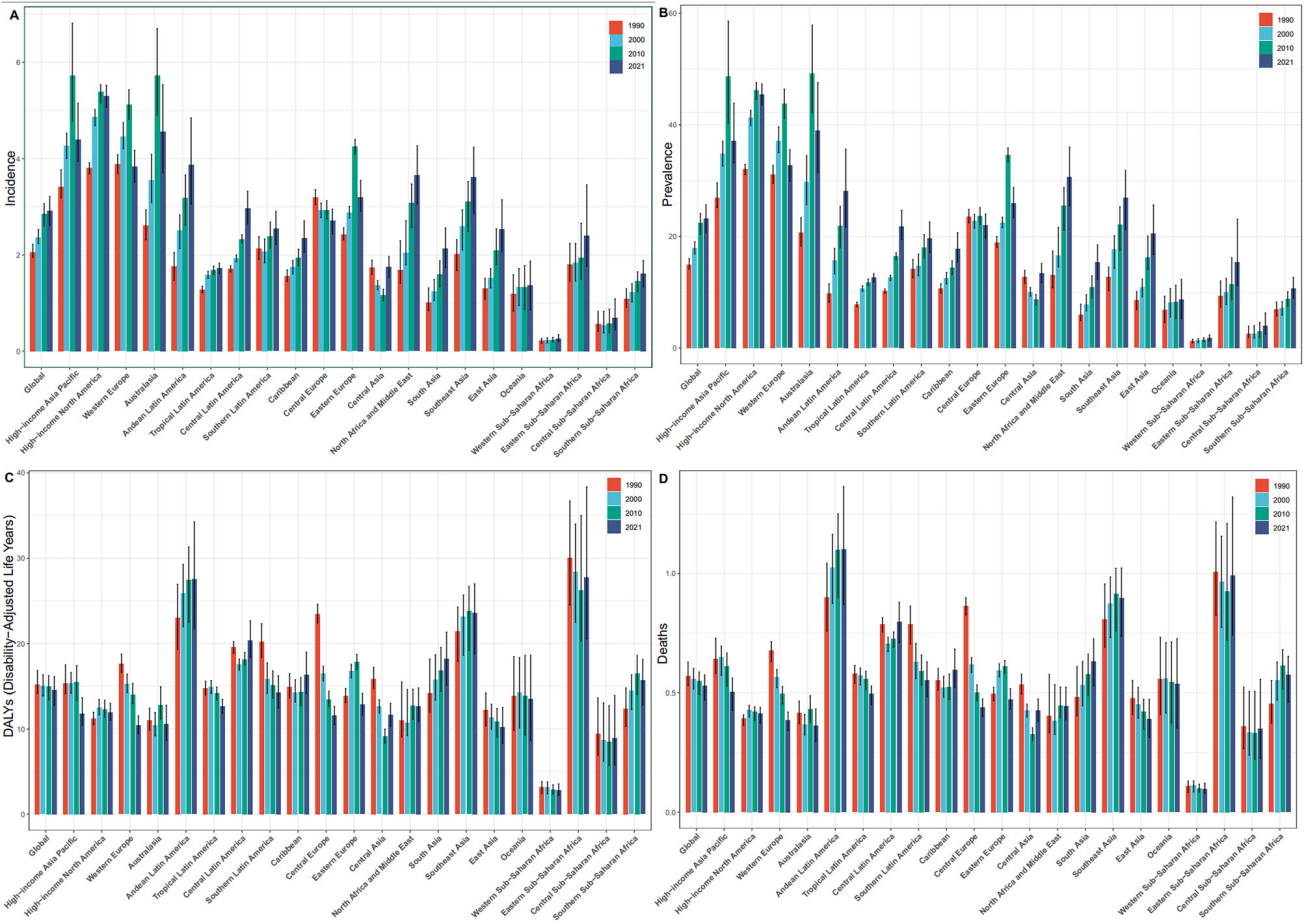
Epidemiologic trends of incidence, deaths, DALYs and prevalence in 21 districts of thyroid cancer from 1990 to 2021. (A). Trends in incidence in 21 districts. (B). Trends of prevalence in 21 districts. (C). Trends of DALYs in 21 districts. (D). Trends of death in 21 districts.

**Figure 6:**
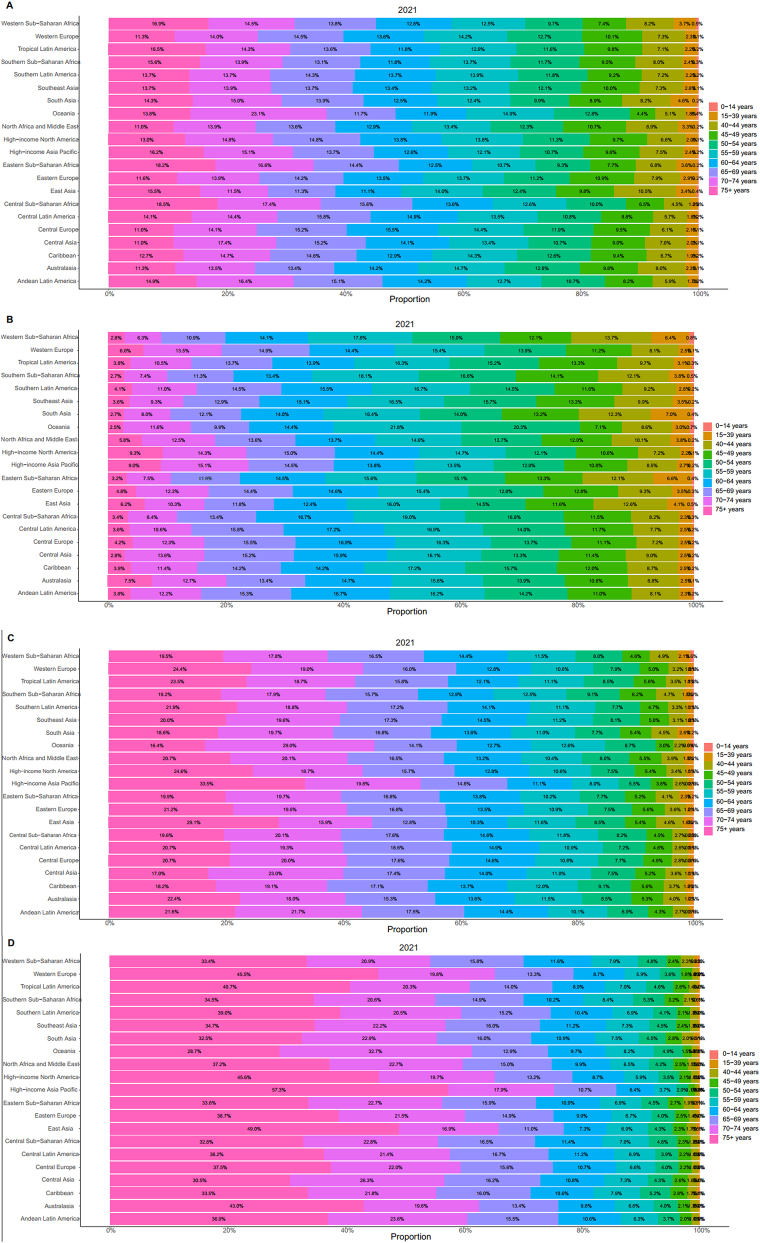
Age distribution of incidence, deaths, DALYs, and prevalence in 21 districts of thyroid cancer in 2021. (A). Age distribution of incidence. (B). Age distribution of prevalence. (C). Age distribution of DALYs. (D). Age distribution of death.

Among the 21 GBD regions, High-income North America and Australasia had the highest incidence rates in 2021, at 5.302 (95 % UI: 5.526–5.075) and 4.566 (95 % UI: 5.543–3.702), respectively. Western Sub-Saharan Africa had the lowest incidence at 0.262 (95 % UI: 0.342–0.201). Overall, nine regions had incidence rates above the global average, whereas 12 regions fell below it. Between 1990 and 2021, the greatest increase in the incidence of thyroid cancer was observed in North Africa and the Middle East (EAPC: 2.889; 95 % CI: 2.715–3.064). Decadal comparisons revealed that the High-income Asia Pacific, High-income North America, Western Europe, Australasia, and Eastern Europe consistently had incidence rates exceeding the global average across all four time points (Figure 4A). Age-specific analysis indicated that more than half of the patients were aged over 60 years (Figure 5A). Furthermore, the incidence rate presented a strong positive correlation with SDI, with higher SDI levels being associated with higher incidence (Figure 6A).

#### Prevalence

High-income North America and Australasia also exhibited the highest prevalence rates, at 45.473 (95 % UI: 47.333–43.567) and 38.926 (95 % UI: 47.573–31.438), respectively. In contrast, Western Sub-Saharan Africa had the lowest prevalence rate, at 1.752 (95 % UI 2.337–1.303). Eight regions reported prevalence rates above the global mean, whereas 13 regions had rates below the global mean. Between 1990 and 2021, the largest increase in thyroid cancer prevalence was observed in Andean Latin America (EAPC: 3.554; 95 % CI: 3.333–3.775). At each decadal time point, prevalence increased in 11 regions, including Andean Latin America and Central Latin America (Figure 4B). Age-specific analysis indicated that most cases were concentrated among patients aged 55–59 years (Figure 5B). Furthermore, prevalence showed a positive correlation with SDI (Figure 6B).

#### DALYs

In 2021, Eastern Sub-Saharan Africa and Andean Latin America had the highest DALYs rate, at 27.756 (95 % UI: 20.555–38.358) and 27.535 (95 % UI: 21.722–34.253), respectively. Conversely, Western Sub-Saharan Africa had the lowest DALY rate, at 2.816 (95 % UI: 2.214–3.549). Seven regions reported DALY rates higher than the global level, while 14 regions were below it. From 1990 to 2021, the greatest decline was observed in Central Europe (EAPC: −2.468; 95 % UI: −2.772 to −2.162). DALYs rates in Andean Latin America and Eastern Sub-Saharan Africa remained consistently high throughout the study period (Figure 4C). Age-specific analysis revealed that patients aged 70–74 years and those aged 75 years or older accounted for the largest proportion of DALYs. Notably, in High-income Asia Pacific, the DALY rate among patients aged ≥75 reached 33.5 % (Figure 5C). No significant correlation was observed between DALYs rate and SDI (Figure 6C).

#### Mortality

In 2021, Andean Latin America had the highest death rate, at 1.101 (95 % UI: 0.871–1.363), whereas Western Sub-Saharan Africa had the lowest death rate, at 0.097 (95 % UI: 0.079–0.122). Overall, nine regions had death rates higher than the global mean, while 12 regions fell below it. The highest reduction in death rates between 1990 and 2021 was observed in Central Europe (EAPC: −1.855; 95 % CI: −2.096- to −1.614). Except in 1990, Andean Latin America consistently exhibited the greatest death rates across the study period. Western Europe, Tropical Latin America, Southern Latin America, Central Europe, and East Asia all demonstrated steady declines in death rates over successive decades (Figure 4D). Age-specific analyses showed patients aged 75 years and older had the highest death rate, reaching 57.3 % in High-income Asia Pacific (Figure 5D). Mortality rates showed no significant correlation with SDI (Figure 6D).

### Thyroid cancer: national trends

#### Incidence

**Figure 7:**
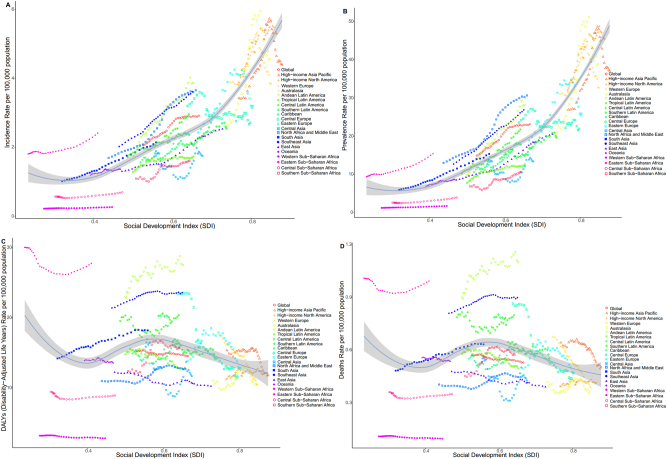
Incidence, death, DALYs and prevalence rates from 1990 to 2021. (A). Incidence rate. (B). Prevalence rate. (C). DALYs rate. (D). Death rate 7D.

Among the 204 countries, Saudi Arabia had the highest incidence, at 7.131 (95 % UI: 9.331–5.395), whereas Tajikistan reported the lowest, at 0.014 (95 % UI: 0.020–0.009). In total, 78 countries recorded incidence rates above the global average, while 126 countries were below it (Figure 7A).

#### Prevalence

The distribution of prevalence across the 204 countries closely mirrored that of incidence. Saudi Arabia reported the highest prevalence, at 60.134 (95 % UI: 79.413–44.685), while Tajikistan had the lowest, at 0.100 (95 % UI: 0.144–0.067). Overall, 66 countries recorded prevalence rates above the global mean, while 128 countries were below it (Figure 7B).

#### DALYs

Ethiopia had the greatest DALY rate, at 44.185 (95 % UI: 67.378–29.767), whereas Tajikistan had the lowest, at 0.125 (95 % UI: 0.173–0.086). The distribution of DALY rate across countries closely resembled that of mortality. In total, 83 countries reported DALY rates above the global average, while 121 countries were below it (Figure 7C).

#### Mortality

Ethiopia had the highest death rate, at 1.597 (95 % UI: 2.361–1.102), while Tajikistan recorded the lowest, at 0.004 (95 % UI: 0.006–0.003). The data showed that countries with the highest death rates were predominantly concentrated in Southeast Africa. Overall, 91 countries had death rates above the global average, while 113 countries were below it (Figure 7D).

### Risk factors

#### Mortality

**Figure 8:**
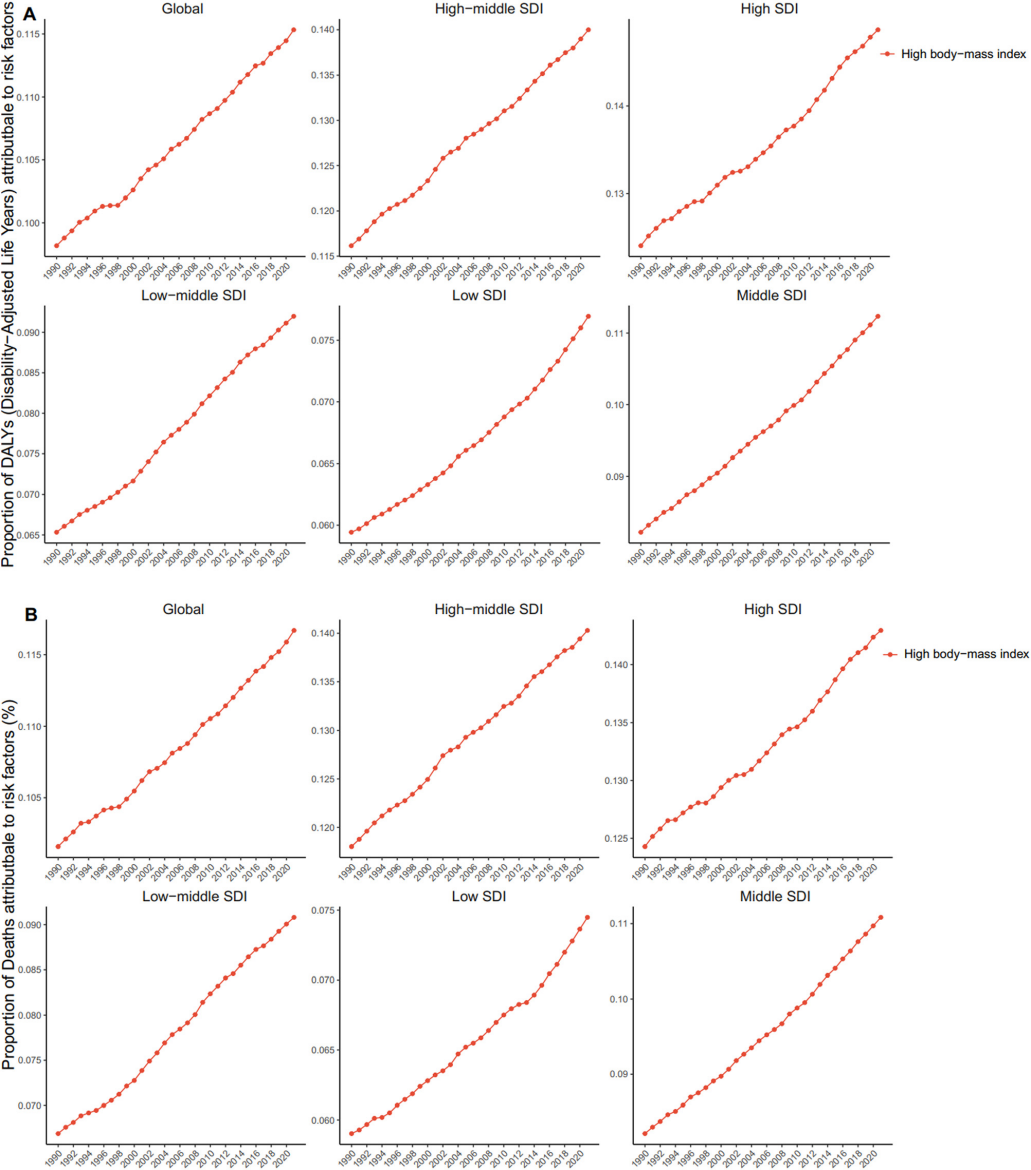
Global trend of risk factors. (A). Global trend in deaths risk factors. (B). Global trend in DALYs risk factors.

**Figure 9:**
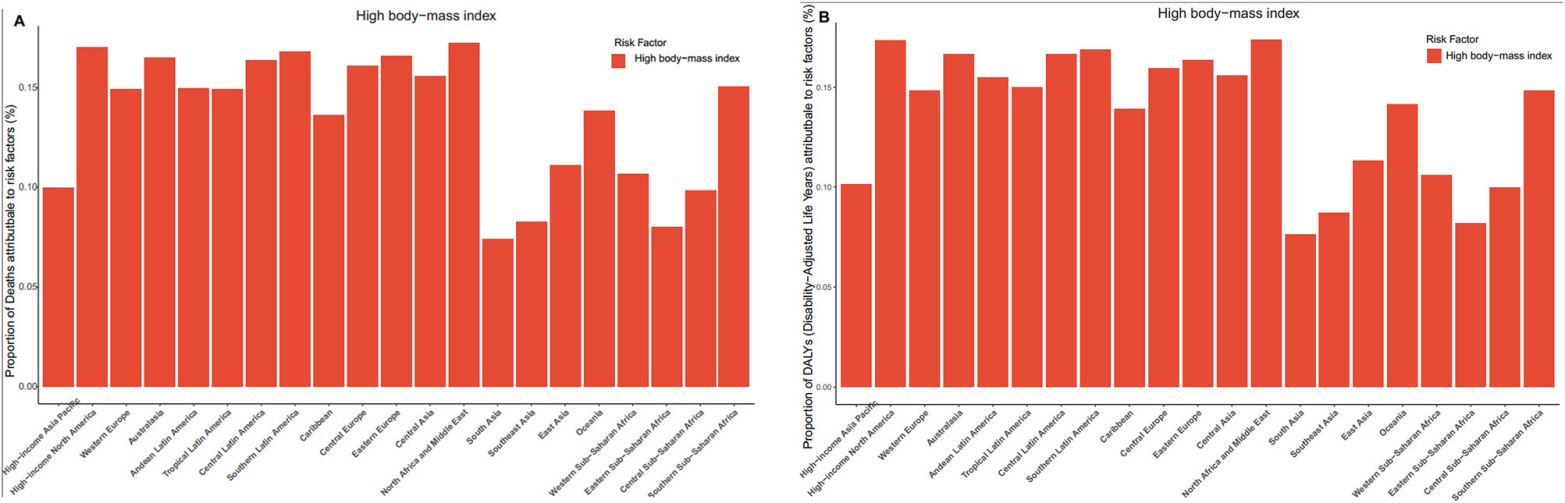
Proportion of risk factors in 21 districts. (A). Proportion of deaths attributed to risk factors in 21 districts. (B). Proportion of DALYs attributed to risk factors in 21 districts.

**Figure 10:**
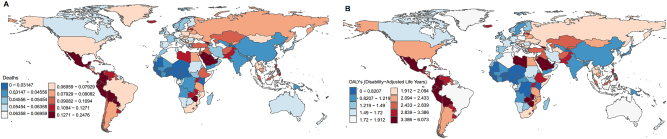
Proportion of global risk factors. (A). Death. (B). DALYs.

**Figure 11:**
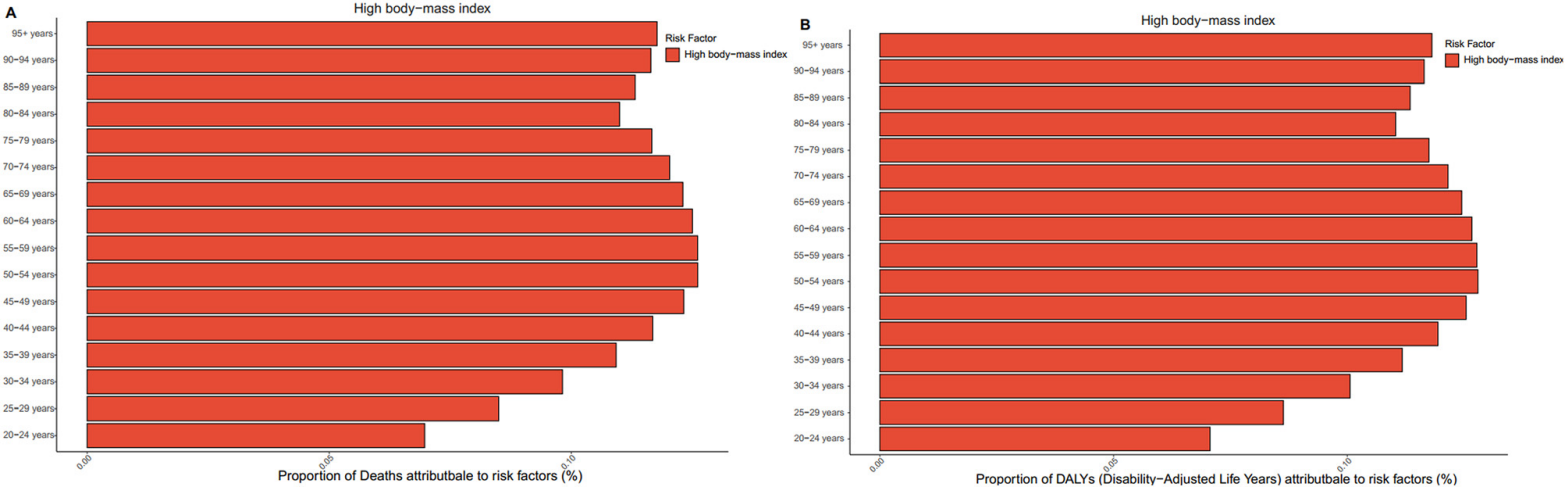
Proportion of risk factors in all age groups. (A). Proportion of deaths attributed to risk factors in all age groups. (B). Proportion of DALYs attributed to risk factors in all age groups.

In 2021, high body mass index accounted for 0.062 % (95 % UI: 0.078–0.046) of global deaths, representing the only risk factor in the GBD analysis. Across SDI regions, high body-mass index contributed most to the death rate in the middle-SDI region (0.063; 95 % UI: 0.081–0.046), and least in the low-SDI region (0.048; 95 % UI: 0.066–0.033). Globally, the proportion of deaths associated with the high body-mass index increased by 0.004 % from 1990 to 2021. A clear association was observed between high body mass index-related death and SDI: the proportion decreased in high-SDI and high-middle-SDI regions, while it increased in middle-SDI, low-middle-SDI, and low-SDI regions (Figure 8A). Regionally, the highest proportion was observed in the Andean Latin America region, where high body mass index accounted for 0.165 (95 % UI: 0.226–0.116) of deaths (Figure 9A). Among the 204 countries, Fiji had the highest contribution of high body mass index to mortality, at 0.248 (95 % UI: 0.359–0.158) (Figure 10A). Analysis across 16 age groups showed that high body mass index had the greatest effect on individuals aged 95 years and older, contributing 1.328 (95 % UI: 1.807–0.779). In contrast, the lowest impact was observed in the 20–24 years age group, with a contribution of 0.005 (95 % UI: 0.007–0.003) (Figure 11A).

#### DALYs

For DALYs, the proportion attributable to high body mass index increased from 1.493 (95 % UI: 1.904–1.124) in 1990 to 1.680 (95 % UI: 2.142–1.264) in 2021. The global and SDI-specific trends in high body mass index-associated DALYs rate closely mirrored those observed for high body mass index-associated death. In the high-SDI region, high body mass index contributed most to DALYs rate (1.749; 95 % UI: 2.198–1.325), whereas the lowest was observed in low-SDI regions (1.381; 95 % UI: 1.912–0.959) (Figure 8B). Across the 21 GBD regions, the highest contribution was recorded in Andean Latin America 4.262 (95 % UI: 5.895–3.030) (Figure 9B). At the country level, Fiji had the highest proportion of DALYs attributable to high body mass index, at 6.07 (95 % UI: 8.985–3.765) (Figure 10B). Age-specific patterns showed that the impact of high body mass index on DALYs rate increased progressively with age. The greatest effect was observed in individuals aged 95 years and older (10.878; 95 % UI: 14.790–6.364), whereas the lowest was seen in the 20–24 years age group (0.350; 95 % UI: 0.504–0.247) (Figure 11B).

## Discussion

Thyroid carcinoma poses a significant and escalating global public health challenge, characterized by a paradoxical increase in incidence alongside declining mortality rates. A comprehensive analysis of global data from 1990 to 2021 reveals complex epidemiological patterns influenced by demographic, socioeconomic, and geographical factors. These findings highlight the urgent need for context-specific public health strategies tailored to the distinct challenges faced by populations across the development spectrum.

The persistent global rise in thyroid cancer incidence (EAPC=1.25), coupled with its striking female predominance (67 % of cases), underscores the synergistic impact of biological susceptibility and healthcare infrastructure. Women’s increased susceptibility to autoimmune thyroiditis is likely attributable to estrogen receptor α (ESR1) signaling and the higher prevalence of the condition [20], 21]. Concurrently, the widespread adoption of sensitive diagnostic technologies, particularly in high-resource settings, has significantly increased the detection of subclinical disease [22], [23], [24], reflecting advances in diagnostic capacity. These trends necessitate rigorous evaluation of the potential of overdiagnosis and overtreatment in high-SDI settings. Notably, 72 % of global cases originate from these regions, despite their populations representing only a minority of the world’s total.

The stark contrast between the distribution of cases (72 % in high-SDI regions) and the mortality burden (68 % in low-SDI regions) underscores the dual nature of the global thyroid cancer challenge. In high-SDI countries, widespread implementation of rigorous screening procedures has resulted in the detection of numerous indolent cancers unlikely to affect morbidity or mortality [25], [26], [27], contributing to the problem of overdiagnosis. Conversely, in resource-limited settings, delayed diagnosis and restricted access to specialized care often result in presentation at advanced stages, when treatment options are limited and outcomes are poor. This epidemiological dichotomy necessitates the implementation of region-specific interventions: de-escalation of surveillance protocols for low-risk areas in high-SDI regions, in conjunction with the concurrent scaling up of early detection programs and improved access to effective treatment in low-SDI settings.

The pronounced national variations in incidence, as exemplified by the contrast between Saudi Arabia (7.13/100,000) and Tajikistan (0.014/100,000), are likely indicative of iodine-mediated pathobiology. Excessive iodine intake in some regions has been demonstrated to promote follicular cell hyperplasia and potentially oncogenic transformation, whereas severe deficiency may paradoxically confer protection against certain thyroid cancer subtypes through different mechanisms [28], [29], [30]. The findings of this study highlight the need for further investigation into population-specific iodine optimization strategies. Such strategies should aim to balance the prevention of iodine deficiency disorders with the mitigation of potential cancer risk.

The disproportionately elevated mortality among elderly males (reaching 12.57/100,000 in those aged 90–94 years) likely reflects a combination of biological and clinical factors. Men are more prone to aggressive histological subtypes, including poorly differentiated and anaplastic carcinomas, and often present at more advanced stages due to delayed healthcare-seeking behavior. This mortality pattern emphasizes the need for age- and sex-specific diagnostic protocols, including the investigation of thyroid nodules in older male patients at lower thresholds and the establishment of rapid referral pathways for this high-risk demographic.

The substantial contribution of elevated BMI to disease burden in specific regions, notably Andean Latin America (4.26 % of DALYs), suggests that adipokine-mediated angiogenesis and insulin resistance are key mechanisms in thyroid carcinogenesis. Activation of the insulin-like growth factor 1 (IGF-1) pathway in obesity has been hypothesized to stimulate thyroid cell proliferation while inhibiting apoptosis [31], [32], [33]. These findings suggest that public health initiatives targeting metabolic syndrome through lifestyle modification could potentially mitigate regional thyroid cancer burden, while substantially addressing other obesity-associated malignancies.

This study represents a substantial advancement over previous epidemiological reports on thyroid cancer, which have frequently been limited to analyses of incidence and mortality within specific countries or regions over relatively shorter timeframes [34], [35], [36], [37], [38]. By leveraging the unprecedented scope and standardization of the Global Burden of Disease (GBD) 2021 study, the present analysis provides a truly global perspective spanning 32 years (1990–2021). The integration of the most recent dataset enables the identification of novel and divergent trends between high-income and low-income regions (Tables S1–2). This suggests a shift in the global landscape of the disease that may reflect disparities in diagnostic capabilities, risk factors, and access to care. A significant novelty of this work is the quantification of the burden of disability-adjusted life years (DALYs), with particular attention to early-onset cases (diagnosed before the age of 50). By combining incidence, mortality, and DALYs, our study provides a more comprehensive perspective, which is critical for informing targeted public health strategies and optimizing resource allocation on a global scale.

It is imperative to acknowledge the limitations of this study when interpreting the findings. The GBD estimates are dependent on multiple data sources of variable quality, particularly in regions lacking comprehensive cancer registries. Furthermore, the analysis of risk factors is constrained to those included within the GBD framework, potentially overlooking other relevant determinants. Finally, the ecological design of the study precludes causal inference at the individual level.

## Conclusions

In conclusion, the present study provides a comprehensive assessment of the global thyroid cancer burden over the past three decades, revealing persistent disparities across gender, age, and socioeconomic groups. These findings underscore the need for two paradigm shifts in thyroid cancer management: first, the adoption of risk-stratified screening approaches in high-SDI regions to reduce overdiagnosis while maintaining early detection of aggressive disease; and second, the strengthening of health systems in the low-SDI regions through scalable early-detection programs and affordable treatment options. Future research should prioritize molecular epidemiology to refine etiological understanding, develop robust prognostic biomarkers, and identify novel therapeutic targets for advanced disease. Targeted, evidence-based interventions will be essential to address the evolving global challenge of thyroid cancer effectively.

## Supplementary Material

Supplementary Material
