# Supplementary material for: Global epidemiology of thyroid cancer: trends in incidence, mortality, and DALYs from 1990 to 2021
Source: Open Med (Wars). 2026 Jan 30;21(1):20251322. doi: 10.1515/med-2025-1322 (PMC12917600; doi:10.1515/med-2025-1322)
Supplement: Supplementary file 1 — Supplementary Material [file j_med-2025-1322_suppl_001.pdf]

TableS1-1 Age-standardized rate incident

location_name	1990-Both	2021-Both	1990-2021EAPC	1990-2021 percent change
Global	2.062(1.951-2.224)	2.914(2.607-3.213)	1.250(1.130-1.370)	0.089(0.003-0.171)
High SDI	3.546(3.434-3.659)	4.493(4.252-4.746)	1.014(0.736-1.293)	0.142(0.092-0.183)
High-middle SDI	2.347(2.189-2.491)	3.068(2.754-3.489)	1.051(0.883-1.220)	0.105(-0.021-0.239)
Middle SDI	1.351(1.212-1.597)	2.711(2.259-3.047)	2.373(2.280-2.466)	0.218(0.029-0.417)
Low-middle SDI	1.047(0.907-1.309)	1.965(1.651-2.339)	2.092(2.067-2.116)	0.581(0.357-0.898)
Low SDI	1.131(0.912-1.422)	1.689(1.328-2.200)	1.229(1.114-1.344)	0.217(0.004-0.526)
Andean Latin America	1.760(1.472-2.050)	3.868(3.052-4.847)	2.604(2.434-2.775)	0.180(-0.078-0.495)
Australasia	2.605(2.320-2.936)	4.566(3.702-5.543)	2.612(2.041-3.186)	0.121(0.044-0.198)
Caribbean	1.563(1.451-1.691)	2.358(2.055-2.704)	1.503(1.313-1.694)	0.092(-0.049-0.233)
Central Asia	1.735(1.602-1.894)	1.748(1.540-1.970)	0.009(-0.757-0.780)	0.423(0.206-0.666)
Central Europe	3.198(3.043-3.356)	2.708(2.443-2.949)	-0.654(-0.864--0.444)	0.140(0.042-0.229)
Central Latin America	1.714(1.651-1.775)	2.963(2.640-3.322)	1.662(1.516-1.807)	0.042(-0.058-0.150)
Central Sub-Saharan Africa	0.568(0.412-0.822)	0.691(0.446-1.086)	0.626(0.414-0.839)	0.038(-0.302-0.508)
East Asia	1.301(1.075-1.517)	2.530(2.070-3.145)	2.429(2.251-2.606)	0.250(-0.079-0.642)
Eastern Europe	2.426(2.314-2.562)	3.202(2.899-3.548)	1.273(0.782-1.767)	0.163(0.060-0.273)
Eastern Sub-Saharan Africa	1.806(1.460-2.241)	2.408(1.767-3.452)	0.760(0.592-0.928)	0.219(-0.013-0.549)
High-income Asia Pacific	3.419(3.185-3.768)	4.396(3.942-5.153)	1.147(0.662-1.634)	0.140(0.052-0.201)
High-income North America	3.812(3.683-3.913)	5.303(5.075-5.526)	1.150(0.965-1.336)	0.095(0.065-0.121)
North Africa and Middle East	1.694(1.407-2.299)	3.653(3.053-4.269)	2.889(2.715-3.064)	0.565(0.283-0.995)
Oceania	1.192(0.834-1.590)	1.363(0.854-1.872)	0.331(0.235-0.426)	0.235(0.004-0.557)
South Asia	1.013(0.842-1.316)	2.136(1.737-2.557)	2.540(2.458-2.622)	0.413(0.094-0.886)
Southeast Asia	2.021(1.678-2.321)	3.615(2.856-4.239)	1.816(1.734-1.899)	0.518(0.242-0.822)
Southern Latin America	2.133(1.910-2.383)	2.541(2.220-2.901)	0.696(0.468-0.925)	-0.029(-0.110-0.052)
Southern Sub-Saharan Africa	1.091(0.912-1.300)	1.612(1.340-1.884)	1.545(1.291-1.799)	0.515(0.279-0.801)
Tropical Latin America	1.285(1.217-1.349)	1.728(1.613-1.831)	0.759(0.596-0.921)	0.236(0.184-0.284)
Western Europe	3.881(3.692-4.082)	3.840(3.512-4.176)	0.284(-0.113-0.682)	0.153(0.102-0.195)
Western Sub-Saharan Africa	0.218(0.161-0.264)	0.262(0.201-0.342)	0.518(0.451-0.584)	0.916(0.603-1.343)

TableS1-2 Age-standardized rate prevalence

location_name	1990-Both	2021-Both	1990-2021EAPC	1990-2021 percent change
Global	14.931(14.124-16.029)	23.143(20.663-25.647)	1.584(1.436-1.732)	0.165(0.073-0.251)
High SDI	28.451(27.601-29.284)	38.161(36.318-40.373)	1.229(0.920-1.539)	0.312(0.253-0.356)
High-middle SDI	17.550(16.387-18.650)	25.224(22.581-28.813)	1.400(1.211-1.589)	0.135(0.003-0.277)
Middle SDI	8.646(7.671-10.104)	21.017(17.457-23.842)	3.031(2.943-3.119)	0.237(0.039-0.448)
Low-middle SDI	6.259(5.348-7.893)	13.952(11.513-16.940)	2.672(2.640-2.703)	0.595(0.362-0.914)
Low SDI	6.151(4.866-7.885)	11.137(8.657-14.931)	1.873(1.754-1.992)	0.221(-0.000-0.535)
Andean Latin America	9.768(8.116-11.497)	28.146(21.632-35.637)	3.554(3.333-3.775)	0.203(-0.060-0.533)
Australasia	20.631(18.285-23.338)	38.926(31.438-47.573)	2.874(2.261-3.492)	0.350(0.254-0.450)
Caribbean	10.635(9.838-11.528)	17.803(15.424-20.630)	1.863(1.680-2.046)	0.152(-0.000-0.298)
Central Asia	12.708(11.596-13.930)	13.377(11.742-15.155)	0.159(-0.641-0.965)	0.398(0.186-0.640)
Central Europe	23.556(22.394-24.813)	21.932(19.706-23.976)	-0.332(-0.533--0.131)	0.160(0.060-0.252)
Central Latin America	10.201(9.827-10.602)	21.782(19.429-24.617)	2.389(2.274-2.505)	0.077(-0.028-0.191)
Central Sub-Saharan Africa	2.596(1.832-3.888)	3.953(2.501-6.286)	1.420(1.127-1.714)	0.049(-0.299-0.546)
East Asia	8.547(6.882-10.101)	20.509(16.769-25.610)	3.163(2.988-3.337)	0.283(-0.063-0.693)
Eastern Europe	18.913(18.064-19.996)	25.905(23.336-28.758)	1.470(0.947-1.995)	0.152(0.042-0.272)
Eastern Sub-Saharan Africa	9.394(7.333-12.029)	15.432(11.201-23.064)	1.475(1.282-1.668)	0.230(-0.015-0.578)
High-income Asia Pacific	26.919(25.136-29.571)	37.095(33.168-43.838)	1.422(0.891-1.955)	0.293(0.186-0.383)
High-income North America	32.051(31.062-32.891)	45.473(43.567-47.333)	1.233(1.040-1.426)	0.184(0.153-0.212)
North Africa and Middle East	13.034(10.804-17.385)	30.674(25.449-35.997)	3.159(2.983-3.335)	0.572(0.284-0.988)
Oceania	6.849(4.524-9.294)	8.636(5.214-12.327)	0.590(0.471-0.709)	0.251(0.012-0.597)
South Asia	5.948(4.894-7.848)	15.332(12.338-18.511)	3.242(3.153-3.330)	0.413(0.091-0.879)
Southeast Asia	12.720(10.279-14.500)	26.880(21.133-31.790)	2.330(2.226-2.434)	0.540(0.257-0.851)
Southern Latin America	14.146(12.585-15.883)	19.678(17.138-22.577)	1.210(0.995-1.425)	0.006(-0.076-0.086)
Southern Sub-Saharan Africa	6.877(5.761-8.227)	10.672(8.908-12.653)	1.795(1.530-2.060)	0.491(0.270-0.768)
Tropical Latin America	7.794(7.384-8.204)	12.593(11.820-13.322)	1.322(1.123-1.522)	0.269(0.217-0.319)
Western Europe	31.069(29.490-32.706)	32.694(29.892-35.548)	0.514(0.075-0.955)	0.406(0.347-0.460)
Western Sub-Saharan Africa	1.234(0.893-1.537)	1.752(1.303-2.337)	1.091(0.999-1.183)	0.917(0.597-1.336)

TableS1-3 Age-standardized rate DALYs

location_name	1990-Both	2021-Both	1990-2021EAPC	1990-2021 percent change
Global	15.206(14.184-16.830)	14.571(12.783-16.115)	-0.140(-0.168--0.112)	0.008(-0.085-0.096)
High SDI	15.381(14.592-16.281)	11.757(10.791-12.773)	-0.767(-0.860--0.674)	0.025(-0.013-0.060)
High-middle SDI	15.569(14.397-16.646)	11.628(10.442-13.018)	-0.987(-1.057--0.917)	0.012(-0.112-0.145)
Middle SDI	14.017(12.761-16.178)	15.218(12.811-16.807)	0.280(0.218-0.343)	0.142(-0.043-0.339)
Low-middle SDI	13.755(11.989-17.150)	16.755(14.349-19.425)	0.669(0.633-0.704)	0.535(0.306-0.849)
Low SDI	17.751(14.570-21.620)	17.976(14.180-23.060)	-0.050(-0.124-0.023)	0.183(-0.035-0.491)
Andean Latin America	23.024(19.325-26.969)	27.535(21.722-34.253)	0.545(0.430-0.659)	0.130(-0.114-0.448)
Australasia	11.046(9.867-12.461)	10.615(8.673-12.782)	0.512(0.194-0.832)	0.001(-0.063-0.066)
Caribbean	14.915(13.667-16.462)	16.344(14.150-18.983)	0.462(0.239-0.685)	0.125(-0.027-0.274)
Central Asia	15.865(14.737-17.217)	11.663(10.329-13.029)	-1.193(-1.837--0.546)	0.350(0.141-0.594)
Central Europe	23.489(22.423-24.619)	11.604(10.503-12.654)	-2.468(-2.772--2.162)	0.073(-0.020-0.161)
Central Latin America	19.538(18.869-20.227)	20.391(18.359-22.673)	0.003(-0.207-0.213)	0.033(-0.069-0.144)
Central Sub-Saharan Africa	9.444(6.945-13.656)	8.915(5.752-13.928)	-0.185(-0.312--0.058)	0.026(-0.321-0.528)
East Asia	12.247(10.382-14.197)	10.253(8.340-12.519)	-0.561(-0.670--0.451)	0.122(-0.185-0.488)
Eastern Europe	13.857(13.108-14.727)	12.867(11.664-14.145)	-0.286(-0.697-0.127)	0.084(-0.019-0.198)
Eastern Sub-Saharan Africa	30.052(24.542-36.695)	27.756(20.555-38.358)	-0.434(-0.558--0.310)	0.192(-0.053-0.539)
High-income Asia Pacific	15.357(14.147-17.485)	11.815(10.450-13.680)	-0.747(-1.002--0.491)	-0.007(-0.074-0.047)
High-income North America	11.231(10.512-11.973)	11.964(11.039-13.029)	0.209(0.104-0.314)	0.013(-0.010-0.035)
North Africa and Middle East	10.992(9.069-15.492)	12.682(10.870-14.795)	0.758(0.612-0.904)	0.437(0.165-0.816)
Oceania	13.903(9.845-18.463)	13.548(8.609-18.629)	-0.099(-0.144--0.055)	0.224(-0.022-0.582)
South Asia	14.205(11.978-18.175)	18.217(15.089-21.353)	0.848(0.811-0.884)	0.350(0.040-0.801)
Southeast Asia	21.408(17.908-24.290)	23.604(18.798-27.013)	0.274(0.203-0.346)	0.449(0.180-0.745)
Southern Latin America	20.253(18.382-22.338)	14.264(12.484-16.208)	-1.017(-1.295--0.738)	-0.059(-0.135-0.017)
Southern Sub-Saharan Africa	12.405(10.313-14.788)	15.667(12.840-18.144)	0.999(0.712-1.286)	0.478(0.261-0.759)
Tropical Latin America	14.784(14.015-15.580)	12.678(11.831-13.486)	-0.651(-0.756--0.545)	0.214(0.163-0.262)
Western Europe	17.602(16.600-18.756)	10.464(9.444-11.538)	-1.501(-1.649--1.353)	0.045(0.008-0.078)
Western Sub-Saharan Africa	3.184(2.411-3.818)	2.816(2.214-3.549)	-0.491(-0.567--0.416)	0.871(0.558-1.287)

TableS1-4 Age-standardized rate deaths

location_name	1990-Both	2021-Both	1990-2021EAPC	1990-2021 percent change
Global	0.570(0.530-0.628)	0.530(0.470-0.575)	-0.232(-0.251--0.213)	0.052(-0.033-0.131)
High SDI	0.586(0.549-0.612)	0.438(0.391-0.467)	-0.876(-0.909--0.843)	0.078(0.029-0.116)
High-middle SDI	0.587(0.544-0.621)	0.434(0.388-0.479)	-0.729(-0.826--0.632)	0.072(-0.048-0.200)
Middle SDI	0.535(0.490-0.630)	0.568(0.484-0.626)	0.115(0.069-0.160)	0.188(0.005-0.380)
Low-middle SDI	0.482(0.419-0.603)	0.598(0.522-0.679)	0.716(0.684-0.747)	0.570(0.351-0.885)
Low SDI	0.599(0.491-0.735)	0.642(0.516-0.799)	0.163(0.113-0.212)	0.223(0.012-0.533)
Andean Latin America	0.899(0.759-1.043)	1.101(0.871-1.363)	0.621(0.544-0.698)	0.164(-0.084-0.462)
Australasia	0.417(0.373-0.466)	0.364(0.296-0.433)	-0.303(-0.535--0.071)	0.043(-0.029-0.114)
Caribbean	0.551(0.511-0.602)	0.597(0.521-0.683)	-0.102(-0.310-0.106)	0.065(-0.071-0.202)
Central Asia	0.533(0.496-0.578)	0.428(0.381-0.475)	-0.900(-1.271--0.528)	0.432(0.213-0.674)
Central Europe	0.864(0.826-0.899)	0.440(0.401-0.475)	-1.855(-2.096--1.614)	0.123(0.026-0.209)
Central Latin America	0.787(0.755-0.815)	0.798(0.710-0.879)	-0.414(-0.563--0.265)	0.021(-0.076-0.125)
Central Sub-Saharan Africa	0.362(0.266-0.524)	0.350(0.226-0.556)	-0.175(-0.249--0.101)	0.038(-0.307-0.509)
East Asia	0.478(0.411-0.552)	0.391(0.314-0.474)	-0.642(-0.698--0.585)	0.192(-0.119-0.561)
Eastern Europe	0.496(0.468-0.523)	0.472(0.432-0.517)	0.446(0.153-0.739)	0.156(0.057-0.263)
Eastern Sub-Saharan Africa	1.009(0.824-1.215)	0.991(0.742-1.320)	-0.207(-0.272--0.143)	0.222(-0.006-0.555)
High-income Asia Pacific	0.642(0.580-0.729)	0.504(0.427-0.561)	-0.677(-0.788--0.565)	0.071(-0.012-0.129)
High-income North America	0.393(0.364-0.409)	0.414(0.375-0.440)	0.119(0.063-0.174)	0.046(0.017-0.073)
North Africa and Middle East	0.405(0.334-0.576)	0.446(0.388-0.523)	0.470(0.369-0.572)	0.516(0.244-0.945)
Oceania	0.557(0.410-0.732)	0.536(0.354-0.727)	-0.068(-0.105--0.032)	0.226(-0.003-0.544)
South Asia	0.483(0.405-0.611)	0.632(0.529-0.726)	0.876(0.832-0.921)	0.414(0.096-0.891)
Southeast Asia	0.809(0.692-0.957)	0.898(0.737-1.023)	0.474(0.409-0.538)	0.502(0.230-0.802)
Southern Latin America	0.786(0.706-0.864)	0.552(0.484-0.630)	-0.965(-1.122--0.808)	-0.047(-0.127-0.035)
Southern Sub-Saharan Africa	0.454(0.374-0.552)	0.574(0.461-0.653)	0.809(0.637-0.980)	0.510(0.272-0.806)
Tropical Latin America	0.580(0.540-0.614)	0.497(0.451-0.528)	-0.636(-0.699--0.574)	0.217(0.166-0.266)
Western Europe	0.677(0.631-0.714)	0.387(0.344-0.420)	-1.616(-1.665--1.567)	0.086(0.039-0.127)
Western Sub-Saharan Africa	0.110(0.084-0.131)	0.097(0.079-0.122)	-0.286(-0.365--0.206)	0.918(0.610-1.349)
